# Understanding short-term transmission dynamics of methicillin-resistant *Staphylococcus aureus* in the patient room

**DOI:** 10.1017/ice.2021.350

**Published:** 2022-09

**Authors:** Aline Wolfensberger, Nora Mang, Kristen E. Gibson, Kyle Gontjes, Marco Cassone, Silvio D. Brugger, Lona Mody, Hugo Sax

**Affiliations:** 1 Division of Infectious Diseases and Hospital Epidemiology, University Hospital Zurich, University of Zurich, Zurich, Switzerland; 2 Division of Geriatric and Palliative Medicine, University of Michigan, Ann Arbor, Michigan, United States; 3 Geriatrics Research Education and Clinical Center (GRECC), VA Ann Arbor Healthcare System, Ann Arbor, Michigan, United States; 4 Department of Infectious Diseases, Bern University Hospital and University of Bern, Bern, Switzerland

## Abstract

**Objective:**

Little is known about the short-term dynamics of methicillin-resistant *Staphylococcus aureus* (MRSA) transmission between patients and their immediate environment. We conducted a real-life microbiological evaluation of environmental MRSA contamination in hospital rooms in relation to recent patient activity.

**Design:**

Observational pilot study.

**Setting:**

Two hospitals, hospital 1 in Zurich, Switzerland, and hospital 2 in Ann Arbor, Michigan, United States.

**Patients:**

Inpatients with MRSA colonization or infection.

**Methods:**

At baseline, the groin, axilla, nares, dominant hands of 10 patients and 6 environmental high-touch surfaces in their rooms were sampled. Cultures were then taken of the patient hand and high-touch surfaces 3 more times at 90-minute intervals. After each swabbing, patients’ hands and surfaces were disinfected. Patient activity was assessed by interviews at hospital 1 and analysis of video footage at hospital 2. A contamination pressure score was created by multiplying the number of colonized body sites with the activity level of the patient.

**Results:**

In total, 10 patients colonized and/or infected with MRSA were enrolled; 40 hand samples and 240 environmental samples were collected. At baseline, 30% of hands and 20% of high-touch surfaces yielded MRSA. At follow-up intervals, 8 (27%) of 30 patient hands, and 10 (6%) of 180 of environmental sites were positive. Activity of the patient explained 7 of 10 environmental contaminations. Patients with higher contamination pressure score showed a trend toward higher environmental contamination.

**Conclusion:**

Environmental MRSA contamination in patient rooms was highly dynamic and was likely driven by the patient’s MRSA body colonization pattern and the patient activity.

Methicillin-resistant *Staphylococcus aureus* (MRSA) is a pathobiont that causes both community-acquired and healthcare-associated infections, including skin and soft-tissue infections, surgical site infections, bacteremia, and pneumonia.^1^ The rate of MRSA among *S. aureus* isolates differs largely between countries: in the European Union it was 16.9% (range, 1%–44%) in 2017^2^ and in North America it was 47% between 1997 and 2016.^3^

MRSA can colonize the nares, groin, axilla, and rectum.^4^ Transmission of MRSA between patients occurs via direct or indirect contact and healthcare worker (HCW) hands are thought to play a major role.^5,6^ Bidirectional transmission pathways exist in the triangle between patients, HCW, and environment.^7^ In recent years, accumulating evidence suggests that contaminated surfaces contribute largely to the transmission of hospital pathogens.^8^ For example, patients admitted to a room previously occupied by an MRSA carrier patient have an increased risk of acquiring the same MRSA strain.^9^ HCW hand contamination with MRSA was as likely after contact with generally examined patient body sites (40%) and high-touch surfaces (45%) in MRSA patient rooms.^10^

Several studies have assessed the magnitude of MRSA contamination in the patient environment. The percentage of positive samples is often high, ranging from 10% to 59% overall.^11–15^ Patient immobility is associated with less environmental contamination.^14^ Nonmedical (eg, bathing) and medical procedures (eg, wound care), MRSA infection (vs carriage status), diarrhea, and MRSA wound colonization result in increased environmental contamination.^11,15,16^ MRSA can persist in the environment for months,^17^ but disinfectants reliably reduce the MRSA burden on inanimate surfaces.^18^

Recently, Mody et al^19^ showed that 10% of hospitalized patient hands were contaminated with multidrug-resistant organisms (MDROs), and 5% were MRSA. In parallel, 29% and 8.5% of hospital rooms were contaminated with MDRO and MRSA, respectively. MDRO contamination increased with a longer presence of patients in the room.^19^ However, little is known about the short-term dynamics of MRSA transmission between patients and their immediate environment. We conducted a real-life microbiological evaluation of MRSA colonization of patients’ body sites and contamination of patient hands and inanimate surfaces in their rooms at short intervals. We hypothesized that patient activity would correlate with environmental contamination and that this contamination would be immediate.

## Methods

### Participants and procedures

This pilot study was conducted from November 2018 to July 2019 in 2 hospitals: the University Hospital of Zurich, Switzerland (hospital 1) and the Michigan Medicine Hospital in Ann Arbor, Michigan (hospital 2).

A convenience sample of 10 adult patients colonized or infected with MRSA who provided oral consent were included. Data regarding age, sex, date of admission, presence of activity of daily living (ADL) impairment, indwelling devices, number of open wounds, presence of MRSA infection, antibiotic and chlorhexidine (CHX) use were collected.

Overall, 4 visits took place, a baseline visit at time 0 and 3 follow-up visits at 90-minute intervals at 90, 180, and 270 minutes. To examine the carrier status of each study patient, swabs were taken at baseline from the body sites groin, axilla, and nares. Each patient’s dominant hand was also sampled for MRSA contamination. Additionally, at hospital 1 and hospital 2, samples for culture were taken from the following 4 high-touch surfaces of each patient’s room: the bedside table, the bathroom inside door handle, the toilet seat, the bed remote control. At hospital 1, the patient’s room inside door handle and the bed rail were also sampled. At hospital 2 additional samples from the television remote control and the room phone were collected. At follow-up visits, sampling included the patient’s dominant hand, the aforementioned high-touch surfaces, and (hospital 1 only) room air. After each sampling, environmental sites and patient hands were disinfected, at hospital 1 with 80% ethanol for hands and inanimate surfaces, at hospital 2 with 70% ethanol for hands and accelerated hydrogen peroxide wipes (Oxivir, Diversy, Fort Mill, SC, USA) for inanimate surfaces. To verify the effect of disinfection, additional hand and environmental samples were taken after disinfection at the baseline visit. Routine cleaning of high-touch surfaces by cleaning staff was postponed until after the study procedures were completed for the day.

At hospital 1, activities in the patient room during the 90-minute time intervals were self-reported by the patient, guided by a semistructured questionnaire (Supplementary Material online). Frequency and duration of patient activities (sleeping, eating, etc), personal care (showering, hand cleansing, etc), therapeutic interventions (wound care, physiotherapy, etc), and the presence of other individuals were assessed. In hospital 2, the activity level of each patient in their room was registered using a centrally placed video camera over the study period, and this information was analyzed manually.

### Microbiological sampling and culturing

Premoistened eSwabs with Amies medium (Copan Diagnostics, Murietta, GA) were used to sample body sites. FLOQSwabs with swab rinse kit (SRK) solution (Copan Diagnostics, Murietta, GA), effective to inactivate disinfecting agents including ethanol, were used to sample environmental sites and hands. Air sampling was performed with an air sampler (MAS-100NT) with an airflow of 500 L in 5 minutes that was placed 1 m from the patient’s bed.

Aliquots of 300 µL Amies medium or SRK solution were plated on CHROMagarMRSA (CHROMagar, Paris, France) within 1 hour after sampling. CHROMagar MRSA has shown a sensitivity of 100% and a specificity of 99%–100% for MRSA after 48 hours of incubation.^20,21^ After incubation at 36°C for 48 hours, colonies consistent with *S. aureus* were tested for coagulase production using BBL Staphyloslide Latex Kit (Becton Dickinson, Franklin Lakes, NJ). The number of MRSA colony-forming units (CFUs) were counted. For all swab samples, enrichment cultures were performed with nonselective enrichment broth and consecutive plating on CHROMagar MRSA. Air sample cultures were performed on Columbia agar with 5% sheep blood (COS) agar, and staphylococcal colonies were tested for coagulase production. MRSA identification was achieved by subculturing colonies on CHROMagar MRSA.

### Contamination pressure score

The patients’ activity level was semiquantified by 2 authors (N.M. and A.W.), and in case of disagreement, a third author (H.S. or K.E.G.) assisted in achieving consensus. A 4-item scale was used to assess activity, ranging from “very inactive” (ie, patient mostly staying in bed) to “very active” (ie, patient mobile, leaving bed several times). The least and most active patients among the 10 included patients represented the reference for classification. The activity score was multiplied by the number of colonized body sites to create a contamination pressure score.

### Data analysis

For this pilot study, we determined the sample size of 10 patients and 40 visits with 240 environmental samples to reasonably represent potential colonization and transmission patterns. Data for both microbiologic results and patient behavior were analyzed descriptively. Ordered logistic regression analysis was used to test for correlation of the contamination pressure score with the sum of all contaminated environmental sites per patient in all 3 follow-up visits. Statistical analyses were performed using STATA version 15 software (StataCorp, College Station, TX).

The necessity for a formal ethical evaluation was waived by the Zurich Cantonal Ethics Commission (no. Req-2018-00007). The study was reviewed and approved by the University of Michigan Institutional Review Board (no. HUM00141575).

## Results

### Participant characteristics

In total, 10 patients (7 female) with a median age of 55 years (range, 26–81) participated in the study (Table 1). All were under contact isolation precautions, and 6 patients received antibiotic treatment with vancomycin for their MRSA infection. All patients at hospital 1 were established MRSA carriers at baseline, with positive cultures of at least 1 body site, but this was the case for only 3 patients (60%) at hospital 2. Of the10 patients, 2 (both at hospital 2) had received CHX bathing in the 3 days preceding the study.

**Table 1.** Patient Characteristics

Characteristics	Hospital 1 (n = 5)	Hospital 2 (n = 5)	Total (n = 10)
Age, median y (IQR)	58 (33–77)	51 (28–72)	55 (31–73)
Duration of stay before sampling, median d (IQR)	7 (5–22)	6 (2–11)	7 (4–12)
Male sex	1 (20)	2 (40)	3 (30)
Active MRSA infection	2 (20)	4 (80)	6 (60)
Receiving antibiotics	4 (80)	5 (100)	9 (90)
Anti-MRSA agent	2 (40)	4 (80)	6 (60)
Some ADL impairment	2 (40)	2 (40)	4 (40)
CVC	3 (60)	3 (60)	6 (60)
Open wound(s)	2 (40)	4 (80)	6 (60)
CHX body wash within past 3 d	0 (0)	2 (40)	2 (20)
MRSA colonization status			
Nares	3 (60)	1 (20)	4 (40)
Groin	4 (80)	2 (40)	6 (60)
Axilla	2 (40)	0 (0)	2 (20)
Any body site	5 (100)	3 (60)	8 (80)

Note. Data are no. (%) unless otherwise specified. Hospital 1, University Hospital Zurich, Switzerland; Hospital 2, Michigan Medicine Hospital in Ann Arbor, Michigan, USA. ADL, activities of daily living; CVC, central vascular catheter; IQR, interquartile range; MRSA, methicillin-resistant *Staphylococcus aureus.*

### Hand, environmental, and air contamination

**Fig. 1.**
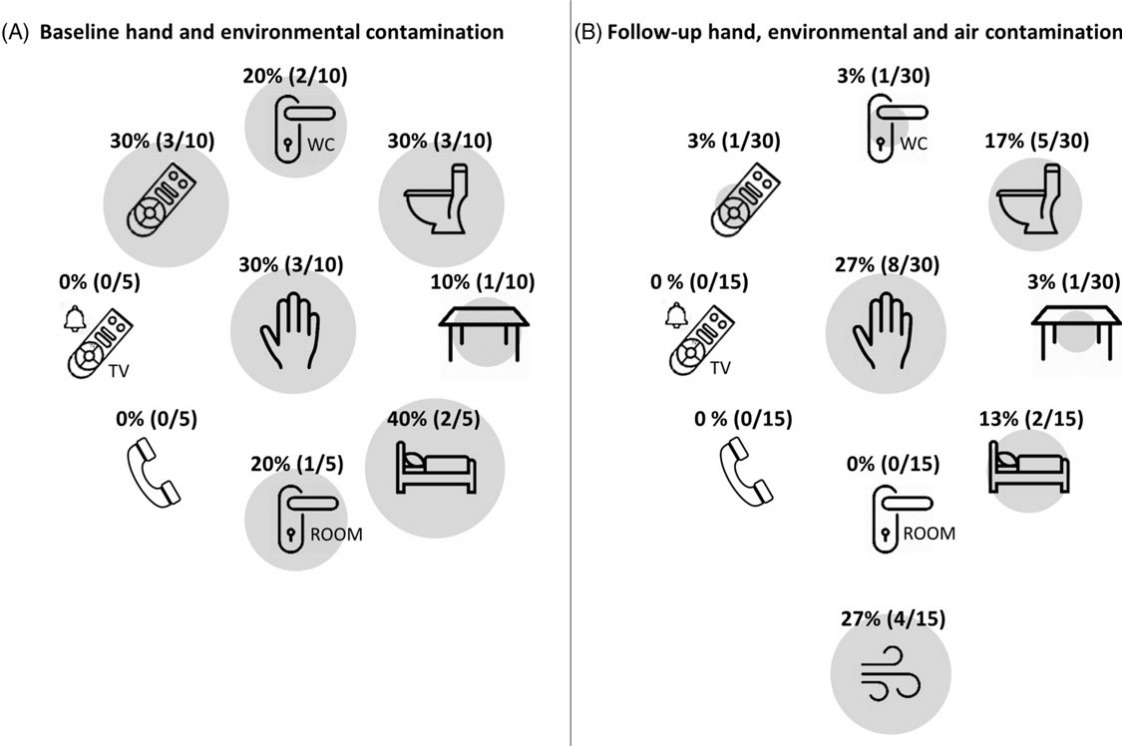
Proportion of MRSA-positive patient hands, environment, and air. Icons represent tested sites, in the center the patient’s dominant hand, in the outer circle the environmental sites: clockwise beginning at 10 o’clock: bed remote control, bathroom inside door handle, toilet seat, bedside table (each for hospital 1 and 2), bed rail and patient room inside door handle (for hospital 1 only), television remote control and room phone (for hospital 2 only), in figure B at the bottom room air. The surface of grey disks are sized proportionately to contamination prevalence of sites, exact prevalence is depicted as a number above.

At hospital 1, the hands of 3 of 5 (60%) patients and 12 of 30 (40%) environmental cultures were MRSA positive at baseline. At hospital 2, no hand nor environmental cultures were positive at baseline. Baseline overall contamination percentages per site are shown in Figure 1a. The median CFU count per sample on contaminated environmental samples was 9 (range, 3–153). All control cultures after disinfection at baseline of patient hands and the high-touch surfaces remained MRSA negative, except for the hand of patient 1 at hospital 1, which grew MRSA in the enrichment culture.

Over the 30 follow-up visits, hands were colonized in 8 (27%) instances (Fig. 1b). Among all 10 patients, 5 (50%) were colonized with MRSA on their hand at least 1 time. Hand cultures were persistently positive at all 3 visits in 1 patient (patient 1) and consistently negative in 5 patients (patients 4, 5, 7, 8, and 10), whereas 4 patients were colonized with MRSA intermittently. Of the 180 environmental swabs during follow-up visits, 10 (5.6%) were contaminated: 9 of 90 (10%) at hospital 1 and 1 of 90 (1.1%) at hospital 2. The environmental site most often identified as MRSA-positive was the toilet seat (17%), followed by the bed rail (13%). The median CFU count per sample was 6 (range, 3–75). Of the 15 air samples at hospital 1, 4 (27%) grew MRSA: 3 in patient 2 and 1 in patient 3. The median CFU count in 500 L air was 7 (range, 2–8).

### Correlation of patient activity and body site colonization with environmental contamination

**Fig. 2.**
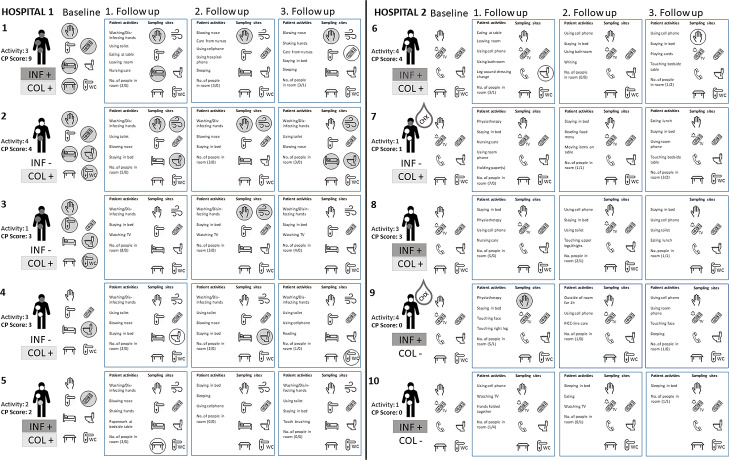
Patient colonization status, hand and environmental contamination, and “contamination pressure” score. Manikin: Patient infected (INF +) or colonized (COL +) with MRSA, shaded circles represent MRSA positive sample sites of nose, axilla and groin (dark grey circles are positive direct cultures, light gray circles are positive enrichment cultures). Drop: Chlorhexidine body wash. Activity: Semiquantification of activity from 1 (ie, very inactive) to 4 (ie, very active). Contamination pressure score: Product of activity score × number of colonized body sites. Items: Sample sites, from upper-left to lower-right corner for hospital 1: patient hand, air, door handle patient room, remote control bed, bed rail, toilet seat, bedside table, door handle bathroom; for hospital 2: patient hand, TV remote control, bed remote control, room phone, toilet seat, bedside table, door handle bathroom; circles represent MRSA-positive sample sites. Grey-colored circles are positive cultures, framed circles are positive enrichment cultures. Patient activities: Description of patient activities during 90-minute episodes; No. of people in room (HCW/non-HCW).

Figure 2 provides a graphic synopsis of the study results including the relevant patient activities during the three 90-minute study intervals. In 23 (77%) of 30 follow-up episodes of our study, nursing or therapeutic procedures took place. A median of 3 (range, 0–8) people entered the room during the 90-minute episodes, of whom a median of 2 (range, 0–8) were HCWs.

The narrative description in Table 2 establishes the potential correlation between patient colonization, hand contamination (as an intermediate vector for high-touch surfaces), and environmental contamination. Patient activity was able to explain 7 (70%) of the environmental contaminations: contamination of all 5 toilet seats and 1 bathroom doorknob was preceded by use of the bathroom by the patient. The bed remote control was contaminated by patient 1 who stayed in bed during the 90 minutes preceding the third follow-up. In the remaining 3 cases (2 bedrails and 1 bedside table), MRSA contamination could have occurred not only from the patient but also from HCWs present in the room during the 90-minute interval.

**Table 2.** Activity-Colonization Matrix With Exemplary Patient Narratives

No. Hospital	No. Patient	Patient or HCW Activities Hypothetically Leading to Hand or Environment Contamination	Colonization Status (Baseline)	MRSA Infection
1	1	Hands: Patient reported blowing nose at FU2 and FU3. No obvious explanation for hand contamination at FU1, possibly self-contamination by touching colonized body parts. Bed rail: Patient left room for an ultrasound procedure before FU1 and was visited by 2 HCW, thus contamination of bed rail could hypothetically be explained by patient or HCW activity. Bed remote control: Patient slept in bed at FU3 but also had visitors. She probably used the bed remote to position herself in the bed.	Nares: 8 CFU/swab Groin: >500 CFU/swab Axilla: 2 CFU/swab	Yes; liver abscess
1	2	Hands: Patient did not report contact of hands with colonized skin, but patient clothes may have served as fomites. Toilet seats: The patient used the toilet at FU1 and FU3, likely leading to direct (via colonized skin) or indirect (via patient hands) seat contamination. Bed rail: The patient was visited by 3 HCW during FU3, for infusion changes and serving lunch. The patient also left her room shortly. The contaminated bed rail could thus be explained either by patient or by HCW activity. Air samples: Positive air samples are hypothetically explained by the high patient and HCW activity throughout the 4.5 hours. Nose blowing happened once in every FU.	Nares: 0 CFU/swab Groin: 92 CFU/swab Axilla: 0 CFU/swab	No
1	3	Hand: Heavily colonized patient who stayed in bed during entire study period but reported to have touched and/or scratched her skin repetitively, thus we hypothesize self-contamination of hand. The patient also reported to have repetitively washed and disinfected her hands throughout the 4.5 hours, driven by the concern to spread MRSA, likely explaining the negative hand samples at FU1 and FU3. Air samples: Patient spent >30 minutes on the phone talking during FU2. Also, 3 HCWs entered the patient room, causing air turbulences hypothetically facilitating MRSA air dispersal.	Nares: 4 CFU/swab Groin: 388 CFU/swab Axilla: >500 CFU/swab	No
1	4	Toilet seats: Patient used the toilet before all FU visits. As the patient was colonized in nares only, contamination of toilet seats might have resulted from touching the seats after nose touching (which was reported by the patient). Bathroom doorknob: Patient used the toilet before FU3 hypothetically contaminating the doorknob with his colonized hands.	Nares: 50 CFU/swab Groin: 0 CFU/swab Axilla: 0 CFU/swab	No
1	5	Bedside table: The patient filled in documents with his doctor at the bedside table at FU1. Thus, the patient might have contaminated the table himself via hands or via coughing, or contamination might have happened indirectly by doctor’s hands after handshake with the patient. The patient reported disinfection of hands presumably explaining negative hand samples.	Nares: 0 CFU/swab Groin: enrichment positive Axilla: 0 CFU/swab	Yes; possible MRSA pneumonia
2	6	Hand: The patient was colonized in the groins and had a wound infection on her leg. Her hand contamination at FU3 may be explained by increased interactions with multiple visitors. Toilet seat: The patient used the bathroom during all 3 FU periods, and contaminated the toilet seat at FU1.	Nares: 0 CFU/swab Groin: >200 CFU/swab Axilla: 0 CFU/swab	Yes; leg wound
2	7	No contamination: This patient, who was weakly colonized in the nares, never showed MRSA on her hands even though her hands were in prolonged contact with her face at all FUs. In total, 12 persons were present in the patient room during the 4.5-hour study period, but no environmental contamination occurred. The weak colonization might be explained by CHX washes before enrollment.	Nares: 15 CFU/swab Groin: 0 CFU/swab Axilla: 0 CFU/swab	no
2	8	No contamination: No hand sample and none of the environmental sites was contaminated, probably as the patient’s hands were not in contact with colonized skin sites. He was recorded to touch his colonized groin area once before FU2; however, no growth was detected on the hand.	Nares: 0 CFU/swab Groin: >200 CFU CFU/swab Axilla: 0 CFU/swab	Yes ; osteomyelitis right foot
2	9	Hand: The hand colonization of this patient without colonization of groin, axilla and nares cannot be assigned to any specific action. The patient had a CHX-body wash 2 days before the study.	Nares: 0 CFU/swab Groin: 0 CFU/swab Axilla: 0 CFU/swab	Yes; MRSA bloodstream infection, several diabetic ulcers, MRSA wound infection with osteomyelitis right leg
2	10	No contamination: The patient had no colonized body sited and was very inactive, mostly sleeping or watching TV. Also, only 2 HCW visits took place.	Nares: 0 CFU/swab Groin: 0 CFU/swab Axilla: 0 CFU/swab	Yes; MRSA bloodstream infection, paraspinal abscess with wound at upper back

Note. HCW, healthcare worker; CFU, colony-forming units; MRSA, methicillin-resistant *Staphylococcus aureus*; FU (FU1, FU2, FU3), follow-up visit (follow-up visit 1, follow-up visit 2, follow-up visit 3).

Video footage data from hospital 2 showed the mean duration of selected patient actions and presence of persons in the patient room per follow-up period (Table 3). Records of all hand contacts with the environment showed that most of the contacts did not lead to MRSA contamination. Video analysis revealed that the TV remote control, the bed remote control, the room phone, the bedside table, and the bathroom door handle were touched a mean of 3.7 minutes, 1.3 minutes, 4.0 minutes, 4.6 minutes, and 0 minutes per 90-minute interval, respectively. In hospital 2, only a single high-touch surface (a toilet seat) was contaminated with MRSA.

**Table 3.** Duration of Hand Contact With Environment, Patient Actions, and Presence of Persons in Patient Room

Variable	All Patients (n = 5), all follow-up episodes (n = 15, 90 minutes each) Mean No. Minutes (SD, Range)
**Object touched by patient**	
Cell phone	22.3 (25.0, 0–74.4)
Own hands	8.3 (9.8, 0–28.4)
Own face/head	5.9 (4.7, 0–18.7)
Bedside table^a^	4.6 (10.8, 0–42.2)
Room phone^a^	4.0 (6.9, 0–24.1)
TV remote/Call button^a^	3.7 (8.3, 0–32.3)
Bed controls/ Bedrail^a^	1.3 (2.5, 0–10.1)
Toilet seat^a,b^	0.9 (2.3, 0–8.3)
Doorknob^a^	0 (0.1, 0–0.3)
**Action performed by patient**	
Sleeping	11.6 (28.0, 0–92.4)
Left room	5.3 (15.9, 0–60.0)
Eating at bedside	4.0 (7.0, 0–22.7)
Playing cards	3.2 (10.3, 0–39.4)
In bathroom	1.2 (2.4, 0–9.1)
**Visitors in room**	
Physical/Occupational therapist	7.9 (17.2, 0–51.7)
Nurse, aide	6.3 (7.7, 0–21.7)
Other employee	3.8 (10.7, 0–41.9)
Nonemployee visitor	1.8 (7.0, 0–27.3)
Physician	0.9 (2.1, 0–7.3)
Food services	0.1 (0.1, 0–0.3)

Note. SD, standard deviation.

a Surfaces cultured at each 90-minute interval.

b Derived from presence in bathroom (no video footage in bathroom)

**Fig. 3.**
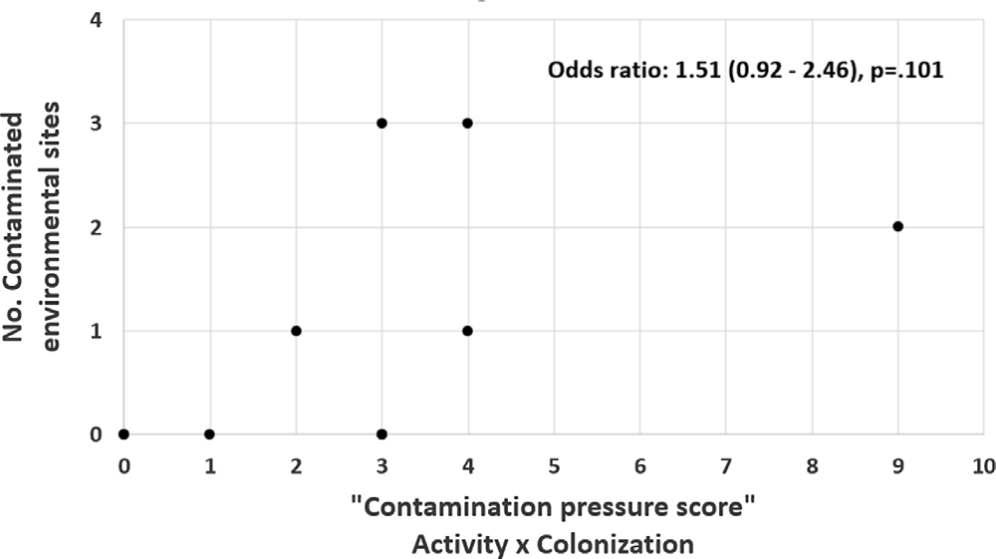
Correlation of contamination pressure score with environmental contamination. The contamination pressure score was calculated by multiplication of “activity level” with the number of colonized body sites (0–3, of axilla, groin, nares). Activity level was defined by quantification of the patient activity (from 1 to 4, with 1 “very inactive,” 2 “inactive,” 3 “active,” and 4 “very active”). Number of contaminated environmental sites are total number of all 3×6 swabbed sites of follow-up 1 to follow-up 3 (ie, theoretical maximum of 18 sites). The odds ratio of the ordered logistic regression analysis is included in the figure.

The median contamination pressure score was 3 (range, 0–9). The contamination pressure scores of individual patients are described in Figure 2. The sum of all contaminated environmental sites of all 3 follow-up visits per patient showed an increasing trend with increasing values of the patient’s contamination pressure score (odds ratio, 1.51; 95% confidence interval, 0.92–2.46; *P* = .10) (Fig. 3).

## Discussion

In our pilot study of 10 patients who were colonized or infected with MRSA, 20% of environmental sites were contaminated with MRSA at baseline. The short-term re-contamination rate within 90-minute intervals, interrupted by decontamination at the beginning of each interval, was 27% for the dominant hand of patients and 6% for environmental sites. Most environmental contaminations were likely explained by the patient’s actions. In contrast, although patients touched their environment often, many of these sites remained free from MRSA contamination.

The dominant hands of 3 patients (30%) tested positive for MRSA at baseline. Despite hand hygiene at the beginning of each successive observation period, in the follow-up visits 27% of hands tested positive, mostly—but not exclusively—in patients who already had baseline hand contamination. Data on hand contamination in patients with MRSA is emerging with 3 studies (including 25–115 patients) describing hand contamination rates between 29% and 82%.^14,22,23^ MRSA carriage on hands can be interpreted as a transient phenomenon,^24^ driven by touching more persistently colonized body sites and, potentially, fomites. Several patients mainly from hospital 1 practiced hand cleansing during the 90-minute study intervals, which potentially contributed to the relatively low rate of hand contamination. Patient hand hygiene has been shown to effectively reduce the burden of MRSA on patient hands,^22^ and it might be effective in reducing MRSA contamination of the patient environment.

We found a low MRSA contamination rate of 6% (10 of 180) of environmental sites after the 90-minute intervals. Rohr et al^14^ identified a comparable low rate of 11% (105 of 1,000) of positive environmental sites in their study, with highest rates in bed linen (24%) and lowest on the wall (1%). Other studies, however, have reported higher rates: Sexton et al^13^ showed a 54% (269 of 502) contamination percentage while including sampling sites like bed linen and mattress, which are in long lasting, close contact with the patient. Boyce et al^15^ reported that 47 (59%) of 80 environmental samples in rooms of patients with diarrhea were MRSA-positive. Alhmidi et al^16^ assessed short-term contamination and found environmental contamination rates after medical and nonmedical procedures compared to a 1-hour period without procedure to be 43% (59 of 138) and 10% (8 of 83), respectively.^16^ In our study including both MRSA infected and colonized patients, nursing or therapeutic procedures took place in 23 (77%) of 30 of follow-up episodes. Alhmidi et al, however, only included patients with MRSA colonization in nares and/or wounds, and they reported a very low rate (3%) of patients on CHX bodywash and a lower rate (31%) of patients on MRSA-active antibiotics, which might explain their higher rates. To our knowledge, the only other study meticulously assessing MRSA transmission by observation of patient and HCW activities was conducted by Ludlam et al. These researchers found that 90% of MRSA transmissions occurred via HCW hands^6^; however, the study setting was an intensive care unit, where patients are usually bedridden.

From the detailed analysis of our 10 patients, 1 of the 2 established main drivers for environmental contamination likely was patient activity. Environmental contamination by the patients themselves was assumed in most cases (7 of 10). A prolonged and intense contact, like going to the toilet, led to the highest contamination percentage. The remainder (3 of 10) of contaminations were not clearly attributable to either patient or HCW. We consider an indirect contamination of patient environment via hands of HCW to be a valid alternative explanation to contamination by patients. A systematic literature review showed that patient care activities lead to HCW glove or hand contamination with MRSA in 11%–58% of activities.^5^

Our study suggests that colonization patterns at the patient level is a further driver of environmental contamination. Low CFU counts or absence of MRSA colonization did not lead to a widespread MRSA contamination of the patient environment (patients 5, 9, and 10). A strong correlation between the number of MRSA-positive body sites and the MRSA contamination of the patient’s room is supported by a study by Rohr et al.^14^ This study including 25 patients in a department of surgery in Germany and showed that 31% samples (75 of 240) taken in rooms of patients with groin colonization were MRSA-positive, whereas only 3.6% (27 of 760) were positive in rooms of patients without groin colonization.^14^

Furthermore, 3 patients in our study had no (patient 9 and 10) or low (patient 5) MRSA colonization. This finding could be related to vancomycin treatment of all 3 patients because MRSA carriage on body sites during vancomycin treatment was shown to be reduced to 40% or below compared to baseline.^6^ Additionally, 1 of the 3 patients (patient 9) was washed with CHX on the day before enrollment. In patients being decolonized with CHX, transmission of MRSA was absent when auditing care episodes,^6^ and the use of CHX was at least independently associated with a lower prevalence of skin and environmental contamination.^23^ CHX body wash, whether as part of a decolonization procedure or as single measure, seems to be effective in preventing MRSA contamination of the hospital room and therefore probably MRSA transmission in the hospital.

In comparison to our low percentages of environmental contamination, we found a rather high percentage of positive air samples (27%). Other authors reported rates of contamination of 4% and 25%,^13,14^ and others found that certain activities, such as bedmaking, led to a higher number of MRSA-containing particles and that movement seemed to be a risk factor for MRSA dispersal.^25^ In our sampling episodes with MRSA identified in the air, we did not find a concomitant contamination of bedside table or bed rail, even though deposition of airborne MRSA can be assumed over time. With a median of 7 CFU of MRSA in 500 L air, the burden might have been too low to relevantly contribute to environmental contamination. On the other hand, the 90-minute intervals may not have been long enough to allow settling of all MRSA-containing particles. Further research is needed to investigate the relevance of airborne MRSA for environmental contamination.

Our study had several limitations. First, it was a pilot study, and only a small number of patients was included. Therefore, we included a range of typical patient profiles in 2 hospitals, each situated in different countries with different MRSA prevalence. Second, we did not perform MRSA typing to prove that environmental and patient isolates contained identical strains. The high colonization dynamics, however, favor in-room spread. Third, on the hand of 1 patient, MRSA was detectable after disinfection, which is in line with the literature, showing that patient hand asepsis often does not completely remove MRSA.^22^

In conclusion, the contamination of patient hands and patient environment is highly dynamic and correlates well with 2 main factors, the number of MRSA colonized body sites and the activity level of the patient. A better understanding of these factors might help to guide infection prevention and control measures, and further research is needed to assess the relevance of these factors (including the contribution of specific MRSA colonized body sites) in a larger, more generalizable patient population. Additionally, CHX body wash and patient hand hygiene are 2 practices to decrease environmental MRSA contamination in patient rooms that deserve further investigation.

## Supplementary material

For supplementary material accompanying this paper visit https://doi.org/10.1017/ice.2021.350. click here to view supplementary material
