# Supplementary material for: Understanding short-term transmission dynamics of methicillin-resistant Staphylococcus aureus in the patient room
Source: Infect Control Hosp Epidemiol. 2021 Aug 27;43(9):1147–54. doi: 10.1017/ice.2021.350 (PMC9272746; doi:10.1017/ice.2021.350)
Supplement: Supplementary file 1 [file S0899823X21003500sup001.docx]

| **Patient activity questionnaire of Hospital 1** | | | | | |
| --- | --- | --- | --- | --- | --- |
| **Action** | **No** | **1x**  **or 10min** | **2-4x**  **or 11-30min** | **≥5x or >30min** | **Notes** |
| **Body care / body contact** | | | | | |
| Showering |  |  |  |  |  |
| Washing total body with soap and water |  |  |  |  |  |
| Washing face with soap and water |  |  |  |  |  |
| Hand washing with soap and water |  |  |  |  |  |
| Hand disinfection with hand rub |  |  |  |  |  |
| Tooth brushing |  |  |  |  |  |
| Using toilet |  |  |  |  |  |
| Blowing nose/touching nose |  |  |  |  |  |
| Skratching skin |  |  |  |  |  |
| Other… |  |  |  |  | Details: |
| **Therapy** | | | | | |
| Physio-/Ergotherapy |  |  |  |  |  |
| Meeting with physicians |  |  |  |  |  |
| Leaving room for procedure |  |  |  |  | Details: |
| Receiving care from nurses |  |  |  |  | Details: |
| Taking medication |  |  |  |  |  |
| Number of HCW in room |  | | | | Number: |
| Other |  |  |  |  | Details: |
| **People contact** | | | | | |
| Shaking hands |  |  |  |  |  |
| Receiving visitors |  |  |  |  |  |
| Number of people in room |  | | | | Number: |
| other |  |  |  |  | Details: |
| **Other patient activity** | | | | | |
| Eating on bedside table |  |  |  |  |  |
| Eating on common table |  |  |  |  |  |
| Staying in bed |  |  |  |  |  |
| Using PC |  |  |  |  |  |
| Using private cellphone |  |  |  |  |  |
| Using hospital phone |  |  |  |  |  |
| Reading book/newspaper |  |  |  |  |  |
| Watching TV |  |  |  |  |  |
| Sleeping |  |  |  |  |  |
| Using the bell |  |  |  |  |  |
| Leaving room |  |  |  |  | Details: |
| Other |  |  |  |  | Details: |
| **Cleaning** | | | | | |
| Cleaning of room by housekeeping |  |  |  |  | Not allowed during study period |
| Cleaning of room by nurses |  |  |  |  |  |
